# Novel Nanostructured Scaffolds of Poly(butylene *trans*-1,4-cyclohexanedicarboxylate)-Based Copolymers with Tailored Hydrophilicity and Stiffness: Implication for Tissue Engineering Modeling

**DOI:** 10.3390/nano13162330

**Published:** 2023-08-14

**Authors:** Giulia Guidotti, Michelina Soccio, Chiara Argentati, Francesca Luzi, Annalisa Aluigi, Luigi Torre, Ilaria Armentano, Carla Emiliani, Francesco Morena, Sabata Martino, Nadia Lotti

**Affiliations:** 1 Civil, Chemical, Environmental and Materials Engineering Department, University of Bologna, 40131 Bologna, Italy; giulia.guidotti9@unibo.it (G.G.); m.soccio@unibo.it (M.S.); 2 Department of Chemistry, Biology and Biotechnologies, University of Perugia, 06123 Perugia, Italy; chiara.argentati@unipg.it (C.A.); carla.emiliani@unipg.it (C.E.); francesco.morena@unipg.it (F.M.); 3 Department of Science and Engineering of Matter, Environment and Urban Planning (SIMAU), Università Politecnica Delle Marche, UdR INSTM, 60121 Ancona, Italy; f.luzi@staff.univpm.it; 4 Department of Biomolecular Sciences, University of Urbino Carlo Bo, Piazza del Rinascimento, 6, 61029 Urbino, Italy; annalisa.aluigi@uniurb.it; 5 Department of Civil and Environmental Engineering, University of Perugia, UdR INSTM, 05100 Terni, Italy; luigi.torre@unipg.it; 6 Department of Economics, Engineering, Society and Business Organization (DEIM), University of Tuscia, UdR INSTM, 01100 Viterbo, Italy; ilaria.armentano@unitus.it

**Keywords:** poly(butylene *trans*-1,4-cyclohexanedicarboxylate), ether linkages, electrospun scaffolds, hydrophilicity, mechanical properties, flexibility, biocompatibility, stem cells culture, adult human multipotent mesenchymal/stromal cells

## Abstract

Here, we present novel biocompatible poly(butylene *trans*-1,4-cyclohexanedicarboxylate) (PBCE)-based random copolymer nanostructured scaffolds with tailored stiffness and hydrophilicity. The introduction of a butylene diglycolate (BDG) co-unit, containing ether oxygen atoms, along the PBCE chain remarkably improved the hydrophilicity and chain flexibility. The copolymer containing 50 mol% BDG co-units (BDG50) and the parent homopolymer (PBCE) were synthesized and processed as electrospun scaffolds and compression-molded films, added for the sake of comparison. We performed thermal, wettability, and stress–strain measures on the PBCE-derived scaffolds and films. We also conducted biocompatibility studies by evaluating the adhesion and proliferation of multipotent mesenchymal/stromal cells (hBM-MSCs) on each polymeric film and scaffold. We demonstrated that solid-state properties can be tailored by altering sample morphology besides chemical structure. Thus, scaffolds were characterized by a higher hydrophobicity and a lower elastic modulus than the corresponding films. The three-dimensional nanostructure conferred a higher adsorption protein capability to the scaffolds compared to their film counterparts. Finally, the PBCE and BDG50 scaffolds were suitable for the long-term culture of hBM-MSCs. Collectively, the PBCE homopolymer and copolymer are good candidates for tissue engineering applications.

## 1. Introduction

Throughout the last decade, tissue engineering and regenerative medicine have been exploring new routes to recreate different types of functional tissues in vitro. To this aim, cells should be seeded onto proper biomaterials and allowed to grow, spread, proliferate, and develop their extracellular matrix (ECM) on three-dimensional (3D) porous substrates, hereafter referred to as scaffolds. These scaffolds must satisfy specific requirements in terms of mechanical properties (e.g., elasticity, stiffness), surface properties (hydrophilicity, micro-/nano-topography, roughness/smoothness), and other chemical–physical characteristics, altogether recapitulating the tissue that must be regenerated. Among these, mechanical properties might influence cell morphology [1,2,3,4,5], surface hydrophilicity, and cell adhesion and proliferation [5]. Therefore, the appropriate modulation of these properties should have a great impact on cell fate.

Of note, the 3D structure of scaffolds fosters the production of a proper architecture of the tissue, and the presence of controlled and adequately interconnected porosity allows cells to permeate into the 3D matrix and growth medium to reach all the cells. In this regard, electrospinning is one of the most common, cost-effective, and easy processing techniques for preparing scaffolds with a suitable microstructure [6].

With regard to the choice of the most appropriate source of polymeric biomaterials to produce these scaffolds, aliphatic polyesters offer several advantages due to their wide range of physical–chemical properties together with high versatility in terms of potential applications. Moreover, most aliphatic polyesters are biodegradable and biocompatible, which are mandatory requirements for applications in biomedicine [7,8,9].

Poly(butylene *trans*-1,4-cyclohexane dicarboxylate) (PBCE) is a biocompatible and easily processable cycloaliphatic polyester, previously investigated for tissue engineering applications by some of the authors of the present paper [10,11,12,13,14]. Although PBCE films have high crystallinity and rigidity characteristics, copolymerization with different amounts of butylene diglycolate (BDG) co-units permits a tunable modulation of the properties (i.e., hydrophilicity, stiffness) without alteration of the biocompatibility of the parent homopolymer [10,15], thus making both the polymer and copolymer attractive for the generation of innovative tunable supports for biological applications.

This study evaluated the effect of the copolymerization (introduction of 50 mol% BDG co-units, referred to hereafter as BDG50, into PBCE homopolymer) in PBCE scaffolds, obtained by electrospinning. This composition was chosen to obtain the best combination of mechanical flexibility, surface hydrophilicity, and cell response, according to our previous works [10,15]. We conducted this study to compare the effect of copolymerization in polymeric films with the same chemical composition and fabricated with the compression molding technique. Due to the different manufacturers, the former has a 3D structure and is highly porous and fibrous, while the latter has a 2D structure and is characterized by a smooth continuous surface. Of note, the chemical modification will have an effect on the surface characteristics and mechanical properties of scaffolds and films. Therefore, it is interesting to explore the biological impact of homopolymer PBCE and copolymer BDG50 scaffolds versus the related film counterparts. To this aim, after a deep chemical–physical characterization, these different biomaterials were tested for protein absorption capability, cell adhesion, proliferation, and long-term cell culture.

As a cell source, we selected adult human multipotent mesenchymal/stromal cells derived from bone marrow (hBM-MSCs) [12,16,17]. Due to their mesenchymal origin, hBM-MSCs, among the types of multipotent/progenitor cells, are considered one of the most appropriate for tissue-engineering-based applications. hBM-MSCs have simple and standardized isolation procedures, immunomodulatory activity, and multipotential properties for in vitro differentiation into non-hematopoietic cell lineages, such as osteocytes, adipocytes, chondrocytes, fibroblasts, and neural cells, using established protocols [18]. 

Here, we demonstrate that a designed copolymerization allows tailoring the hydrophilicity and stiffness of the BDG50 scaffolds without affecting the fibers’ nanostructure. Moreover, PBCE and BDG50 scaffolds are suitable for long-term culture of adult human multipotent mesenchymal/stromal cells.

## 2. Materials and Methods

### 2.1. Materials

*Trans*-1,4-cyclohexanedicarboxylic acid (CHDA, Fluorochem, Glossop, UK), diglycolic acid (DGA, Merck, Darmstadt, Germany), 1,4-butanediol (BD, Merck, Darmstadt, Germany), and titanium IV butoxide (TBT, Merck, Darmstadt, Germany) were all used as purchased.

### 2.2. Synthesis 

**Scheme 1:**
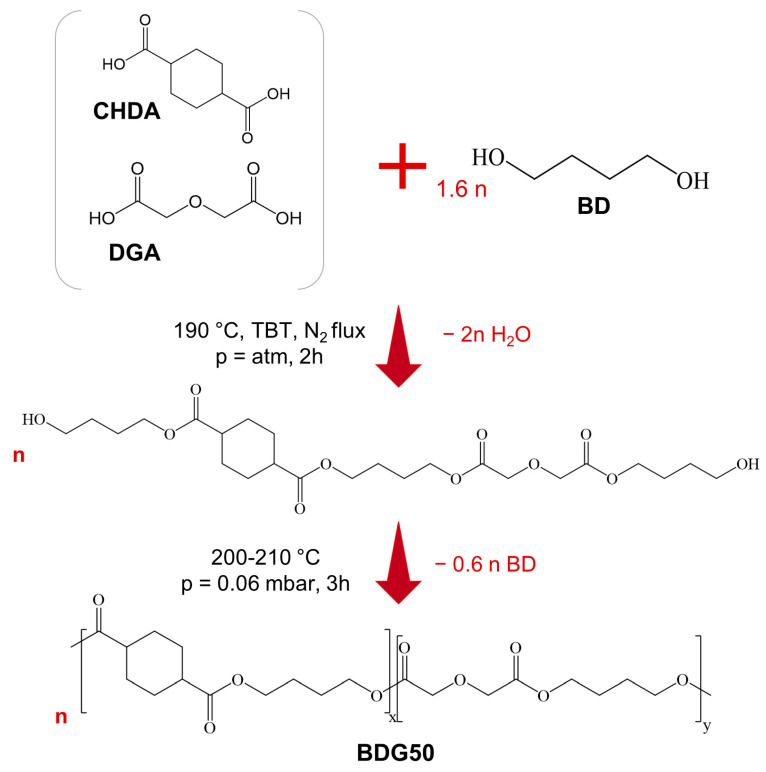
Representation of the synthetic procedure of BDG50 random copolymer.

The syntheses of poly(butylene *trans*-1,4-cyclohexanedicarboxylate) homopolymer (PBCE) and poly(butylene *trans*-1,4-cyclohexanedicarboxylate/diglycolate) random copolymer containing a 50:50 molar ratio of the two diacids were carried out in bulk using a two-stage melt polycondensation reaction according to the procedure already reported in the literature [10], with some modifications, as shown in Scheme 1. Briefly, proper amounts of CHDA (0.22 mol, 28.1 g) and BD (0.352 mol, 31.7 g) in the former case and CHDA (0.12 mol, 15.4 g), DGA (0.12 mol, 16.1 g), and BD (0.384 mol, 34.6 g) in the latter were added, together with the catalyst TBT (about 400 ppm/g of theoretical polymer), to a 250 mL glass reactor under stirring (100 rpm) and placed in a thermostatic silicon oil bath. A glycolic 60% molar excess with respect to the diacid counterpart was employed. In the first stage, the system was maintained in an inert N_2_ atmosphere and the temperature was raised to 190 °C and maintained until 90% of the theoretical amount of water was distilled off (2 h) into a glass condenser connected to the reactor. Then, the second stage started: the temperature was set to 200 °C and a gradual vacuum was applied, up to 0.06 mbar, to promote polycondensation reactions as well as the removal of glycolic excess. During this phase, the torque value became progressively higher, indicating the increase in the molecular weight of the polymeric chains. During the last 30 min, the temperature was raised up to 210 °C. The synthesis was stopped when a constant value of torque was reached and no more distillation was observed (about 3 h in total).

The obtained polymers were then purified through solubilization in chloroform and further precipitation in cold methanol.

### 2.3. Scaffold and Film Preparation

The preparation of electrospun mats was achieved by solving PBCE and BDG50 in 1,1,1,3,3,3-hexafluoro-2-propanol (HFIP) at a concentration of 15% *w*/*v* to obtain 4 mL of solution for each sample. The solutions were then loaded into a syringe (5 mL capacity) connected to a needle (internal diameter of 0.8 mm) and electrospun with a flow rate of 1.8 mL/h. A voltage of 20 kV and a working distance of 15 cm were used for all the samples.

Polymeric films of about 100 μm thickness were obtained through compression molding, using a Carver C12 laboratory press. The polymers were placed between two Teflon sheets at a temperature about 40 °C higher than the melting temperature of each sample. When the polymers were completely melted, a pressure of 5 ton/m^2^ was applied for 2 min and then the films were ballistically cooled to room temperature inside the press. 

### 2.4. Scaffold and Film Characterization

#### 2.4.1. Molecular Characterization

PBCE and BDG50’s chemical structure was determined by means of proton nuclear magnetic resonance (^1^H-NMR) spectroscopy at room temperature, using a Varian Inova 400-MHz instrument. For this, 100 repetitions with a relaxation delay of 0 s and acquisition time of 1 s were performed. Before the measurements, polymeric solutions were prepared using deuterated chloroform containing 0.03 v% tetramethylsilane (TMS) to obtain a concentration of 10 mg/mL.

The same technique was also used to determine the chemical composition of the copolymer and the percentage of *cis* of the 1,4-cyclohexandicarboxylate subunit. In detail, the former has been evaluated from the normalized areas of the ^1^H-NMR resonance peaks related to protons of cyclohexane ring subunit and protons of diglycolic acid, while the latter was calculated from the ratio between the a*_cis_* and a*_trans_* signals areas. 

Number molecular weight (M_n_) and polydispersity index (Ð) were measured through gel-permeation chromatography (GPC) at 30 °C using chloroform solutions (with a concentration of about 2 mg/mL) on HPLC 1100 apparatus (Agilent Technologies, Santa Clara, CA, USA) equipped with a PLgel 5-mm MiniMIX-C column and an RI detector. The calibration curve was recorded using monodisperse polystyrene standards in the range of 2–100 kDa. The eluent flow was 0.3 mL/min. 

Fourier transform infrared spectroscopy (FT-IR) spectra of the PBCE and BDG50 film and membrane were obtained at room temperature (RT) in reflection mode through attenuated total reflectance (ATR) with an FT-IR spectrometer (Jasco FT-IR 615, Tokyo, Japan). The scanned wavenumber range was 4000–600 cm^−1^, at 4 cm^−1^ of spectral resolution.

#### 2.4.2. Morphological Characterization 

The morphology of the electrospun membrane and the film surface of the PBCE and BDG50 was examined using a field emission scanning electron microscope (FESEM Supra 25, Zeiss, Oberkochen, Germany). Samples were cut, and the surfaces were analyzed after gold coating using an Agar automatic sputter coater. Electrospun fibers’ mean diameter and diameter distribution were obtained from the analysis of the corresponding FESEM images using ImageJ software. At least 150 individual fibers were analyzed, and the diameter of each was measured to obtain a reliable result. Data measurement is reported as mean ± SD. 

#### 2.4.3. Surface Characterization

Static water contact angle (WCA) measurements were carried out at room temperature on polymeric films and scaffolds by means of a KSV CAM101 instrument. The side profiles of the deionized water drop (4 μL) were recorded and analyzed immediately and 180 s after drop deposition using Drop Shape Analysis software. The deposition of at least eight different drops was performed on different surface areas, and WCA values were reported as the average value ± standard deviation. 

#### 2.4.4. Thermal Characterization

Thermogravimetric analysis (TGA) was performed on scaffolds and films under inert N_2_ flow (40 mL/min) using PerkinElmer TGA4000 apparatus, heating weighed samples of about 5 mg from 40 up to 800 °C at a rate of 10 °C/min. The temperature corresponding to the onset of the weight loss curve T_onset_ and the temperature of the maximum degradation rate T_max_ were measured.

Calorimetric analysis was performed on scaffolds and films using a PerkinElmer DSC6 instrument under inert N_2_ flow (20 mL/min). Weighed samples of about 8 mg were heated from −60 °C to 180 °C at a rate of 20 °C/min (I scan), held there for 3 min, and then rapidly cooled (100 °C/min) to −60 °C. After that, another heating scan (II scan), carried out under the same conditions as the I scan, was performed. The glass transition temperature (T_g_) was calculated as the temperature corresponding to the midpoint of the glass-to-rubber transition step, visible as an endothermic baseline deviation, and the relative heat capacity increment (Δc_p_) as the step height. The melting temperature (T_m_) and crystallization temperature (T_cc_) were determined as the peak maximum/minimum of the endothermic and exothermic phenomena in the DSC profiles, respectively, while their relative heat of fusion (ΔH_m_) and heat of crystallization (ΔH_cc_) were calculated from the total areas associated with these exothermic and endothermic phenomena, respectively.

#### 2.4.5. Mechanical Characterization

Both the polymers, in the form of films and scaffolds, were characterized from the mechanical point of view through tensile stress–strain measurements by means of an Instron 5966 dynamometer equipped with a transducer-coupled 10 kN loading cell. At least six specimens (50 × 5 mm^2^) for each sample were tested, after having measured their average thickness. The specimens were fixed to the instrument, keeping a gauge length of 20 mm, and then a strain rate of 10 mm/min was applied up to break. The value of elastic modulus (E) was obtained from the initial linear slope of the stress–strain curve, while stress (σ_B_) and elongation at break (ε_B_) were measured as the values of stress and elongation at the breaking point, respectively. The obtained results are reported as mean value ± standard deviation.

### 2.5. Protein Adsorption

The protein adsorption assay was performed using our method [19,20]. Briefly, all films were cut into 0.5 cm^2^ square-shaped pieces and incubated with 2 mg/mL of bovine serum albumin (BSA, Sigma-Aldrich, St. Louis, MO, USA), 2% and 10% of fetal bovine serum (FBS, Euroclone S.p.A, Pero (MI), Italy), and plasma from adult donors at a dilution of 1:10 donors (5 mg/mL) for 30 min and 24 h at 37 °C. The total adsorbed protein content was assessed with the Bradford method [21,22] following three rounds of washing with deionized water (dH_2_O). The absorbance (595 nm) was measured with a microtiter plate reader (GDV-DV990BV6, Roma, Italy). Each sample was analyzed in three independent experiments. Data are reported as mean ± standard deviation.

### 2.6. Culture of Cells on PBCE and BDG50 Films and PBCE and BDG50 Scaffolds

#### 2.6.1. Adult human Multipotent Mesenchymal/Stromal Cells Culture

Adult human multipotent mesenchymal/stromal cells were isolated from bone marrow (hBM-MSCs) and cultured as described in our previous works [12,16,19,23]. The procedure was sporadic and carried out in accordance with the Declaration of Helsinki. The mesenchymal phenotype was analyzed by measuring the expression of markers CD45, CD73, CD90, and CD105 (all from BD Biosciences, San Jose, CA, USA) using the flow cytometer FACScan (BD Biosciences, San Jose, CA, USA) and the FlowJo software (Tree Star, Ashland, OR, USA) for data analysis as previously described [12,16,19,23].

hBM-MSCs were cultured by plating in culture flasks in DMEM high-glucose (Euroclone S.p.A, Pero (MI), Italy) supplemented with 10% fetal bovine serum (FBS, Euroclone S.p.A, Pero (MI), Italy), 1% penicillin-streptomycin (Euroclone S.p.A, Pero (MI), Italy), and 2 mM L-glutamine (Euroclone S.p.A, Pero (MI), Italy) in a humidified atmosphere and 5% of carbon dioxide (CO_2_) at 37 °C (growth culture medium). The medium was changed every three days.

#### 2.6.2. Scaffold and Film Sterilization and Cell Seeding

Scaffolds and films were cut into 1 cm^2^ or 0.5 cm cm^2^ squares, sterilized for 30 s through immersion in 70% ethanol, washed with sterile PBS, and then placed on multi-well plates. After drying, a suspension of 3 × 10^3^ stem cells was seeded drop by drop on sterile materials, and after 45 min, the growth culture medium was gently added. hBM-MSCs on PBCE and BDG50 films and PBCE and BDG50 scaffolds were incubated under canonical culture conditions in a humidified atmosphere at 37 °C, 5% CO_2_. Every three days, the culture medium was changed, and cultures were examined according to the study design.

#### 2.6.3. Cell Proliferation

Stem cells’ proliferation was evaluated by seeding 3 × 10^3^ cells on PBCE and BDG50 films and scaffolds (0.5 cm^2^ squares placed in a 48-well plate) at different time points: 3, 7, 14, and 21 days (D3, D7, D14, and D21). As the internal control, experiments were performed seeding the same number of stem cells on TCP.

Stem cell proliferation was assessed using the Invitrogen^TM^ Countess™Automated Cell Counter (Thermo Fisher, Invitrogen^TM^, Grand Island, NY, USA) according to the manufacturer’s recommendation for adherent cell counting. The use of trypan blue solution, 0.4% (InvitrogenTM, Grand Island, NY, USA), was recommended in the method.

#### 2.6.4. Cell Viability Assay

The viability of hBM-MSCs on scaffolds and films was evaluated by using the MTT (3-(4,5-dimethylthiazol-2-yl)-2,5-diphenyltetrazolium bromide) assay (Sigma-Aldrich) according to the manufacturer’s recommendation. For this, 3 × 10^3^ cells were seeded on 0.5 cm^2^ film and scaffold squares (placed in a 48-well plate) and tissue culture polystyrene (TCP) as the internal control. The effects of each polymeric film square without cells on the MTT test were also taken into consideration. The assay was performed at 3D, 7D, 14D, and 21D in culture. The absorbance was measured using an ELISA reader (microtiter plate reader, GDV-DV990BV6, Roma, Italy) at 589 nm with a reference wavelength of 650 nm. Triplicates of each experiment were run.

#### 2.6.5. Immunofluorescences

Immunofluorescence inspections were carried out as previously described [12,17,19,20,24]. Cultures of cells on films and scaffolds, and on glass coverslips (GC) as an internal control, were rinsed twice with PBS, fixed in 4% paraformaldehyde for 20 min, and then permeabilized (PBS + 3% FBS + 0.5% Triton X-100) and blocked (PBS + 3% FBS + 0.05% Triton X-100) for 1 h at room temperature (RT). Samples were incubated with phalloidin (Alexa-fluor-488 phalloidin, Invitrogen, Grand Island, NY, USA) for 20 min at RT to achieve F-Actin staining or overnight at 4 °C with primary human antibody anti-β-Tubulin (Elabscience, Houston, TX, USA) followed by incubation with secondary antibody conjugated with Alexa-Fluor-594 (Invitrogen™, Grand Island, NY, USA) for 1 h at RT. Samples were mounted and nuclei stained using Vectashield Antifade Mounting Media with 4′,6-diamidino-2-phenylindole (DAPI) (Vector Laboratories Inc., Burlingame, CA, USA) after being washed with PBS. The immunofluorescence was performed at D14 and D21 to assess the biocompatibility of materials in human multipotent cell long-term culture. Image acquisition was performed by using a fluorescence microscope (Eclipse-TE2000-S, Nikon, Tokyo, Japan) equipped with an F-ViewII FireWire camera (Cell^f^ Soft Imaging System, Olympus, Germany, version 2.5, Accessed in 2006). The interference of films and scaffolds without cells was also evaluated.

#### 2.6.6. FESEM Analysis of hBM-MSCs on Scaffolds

Cell and scaffold interaction was evaluated using FESEM. Samples were rinsed twice with PBS and fixed in 2.5% glutaraldehyde for 30 min at RT, then dehydrated by adding progressively more concentrated ethanol (5–100% *v*/*v*) every 5 min, and finally dried under exposure to a Critical Point Machine (CPD, Emitech K850). Once dried, the samples were gold sputter-coated before examination using FESEM (Supra 25 Zeiss) at an accelerating voltage of 5 kV.

#### 2.6.7. Statistical Analysis 

Data analysis is reported as mean ± SD. A post hoc comparison test was carried out using one-way ANOVA and Dunn’s multiple comparison test (GraphPad 6.0 Software, San Diego, CA, USA). *p* < 0.05 was considered statistically significant. 

## 3. Results and Discussion

**Figure 1:**
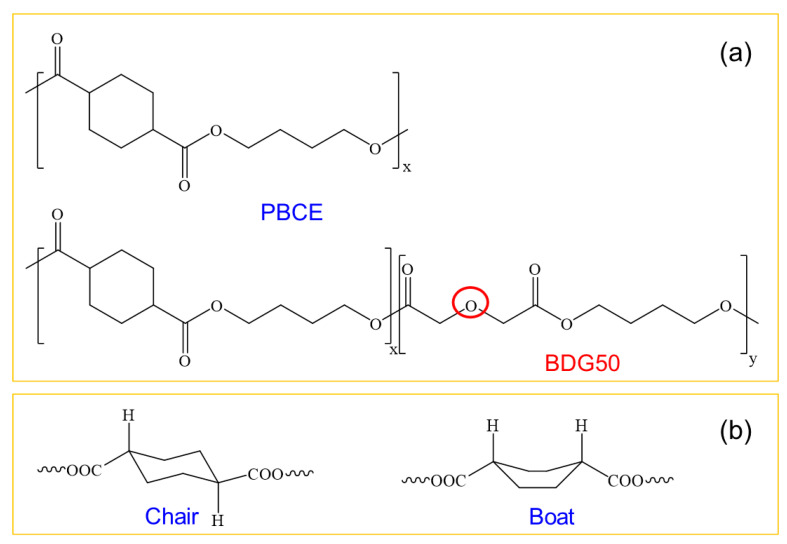
(**a**) Chemical structure of PBCE and BDG50 copolymer; (**b**) *trans* (chair) and *cis* (boat) configurations of the cyclohexane ring.

In this work, we present the design, production, chemical–physical characterization, and biological evaluation of new electrospun scaffolds obtained using two different aliphatic polyesters, poly(butylene *trans*-1,4-cyclohexanedicarboxylate) homopolymer, PBCE, and its random copolymer poly(butylene cyclohexanoate/diglycolate), BDG50 (Figure 1a). The copolymer differs from its parent homopolymer in terms of the presence of ether oxygen atoms in its main chain, which are known to increase the hydrophilicity and flexibility of the final polymer [25,26,27,28]. In addition, an aliphatic cyclohexane ring can adopt two configurations: *trans* (chair conformation) and *cis* (boat conformation) (Figure 1b); the former is characterized by higher symmetry than the latter, resulting in a higher crystallization capability of the *trans* configuration, which forms crystals with a high degree of perfection [10,29].

We compared the properties of PBCE and BD50 scaffolds with those of PBCE and BDG50 films obtained through compression molding techniques and already used by our group [10,12,14].

**Figure 2:**
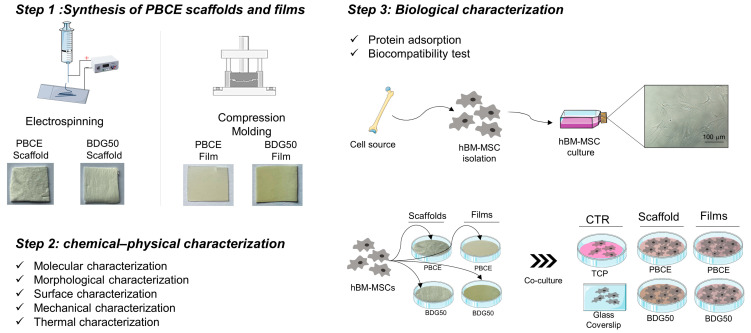
Experimental plan. Step 1: PBCE and BDG50 scaffolds and film synthesis. Step 2: chemical–physical characterization of PBCE and BDG50 scaffolds and films. Step 3: Biological characterization: protein adsorption and biocompatibility test; long-term culture of hBM-MSC on PBCE and BDG50 scaffolds and films; cells on TCP/GC were used as the internal control (CTR).

The experimental plan of this study is reported in Figure 2. Step 1 includes the synthesis of PBCE and BDG50 scaffolds and films; Step 2 refers to the chemical–physical characterization of PBCE and BDG50 scaffolds and films; Step 3 refers to the biological characterization of PBCE and BDG50 scaffolds and films, including the protein adsorption capability, proliferation, viability, adhesion, and morphology of hBM-MSCs. 

### 3.1. Synthesis and Characterization of Scaffolds and Films

At room temperature, both the synthesized polyesters appeared as semicrystalline light yellow solids, the copolymer being more flexible to the touch. After purification, PBCE appeared as white flakes, while BDG50 precipitated as a light yellow rubbery material, lighter than before purification.

#### 3.1.1. Molecular Characterization

**Figure 3:**
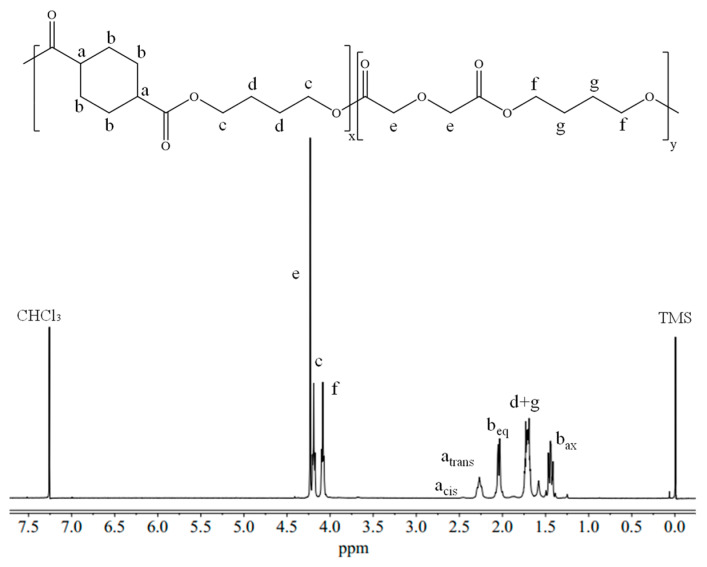
^1^H-NMR spectrum of PBDG50 copolymer with the corresponding peak assignments.

The molecular characterization data of PBCE and BDG50, obtained through ^1^H-NMR and GPC analyses, are listed in Table 1. In Figure 3, the ^1^H-NMR spectrum of the BDG50 copolymer is shown as an example, together with the peaks’ assignment. Apart from the singlets related to the residual CHCl_3_ (7.26 ppm) in deuterated solvent [30,31,32] and the reference TMS (0 ppm), the peaks of both the BCE and BDG co-units are present. 

The cyclohexane ring peaks, a*_trans_* (2H; m; J = 11.3 Hz), a*_cis_* (2H; m; J = 11.1 Hz), b_ax_ (4H; t; J = 12.1 Hz), and b_eq_ (4H; d; J = 10.8 Hz), can be found at δ 2.25, δ 2.45, δ 1.48, and δ 2.10 ppm, respectively. The singlet e (2H; s) related to DGA can be observed at 4.24 ppm. With regard to the BD, the c (4H; t; J = 5.7 Hz), f (4H; t; J = 6.8 Hz), g (4H; m; J = 6.8 Hz), and d (4H; m; J = 5.7 Hz) peaks, these two last partially overlapped, are located at δ 4.19, δ 4.12, and in the range between δ 1.65 and 1.75 ppm, respectively. With regard to the actual BDG50 molar composition, it was found very close to the feed one (Table 1).

As reported in the literature [29], the 1,4-cyclohexylene ring can undergo isomerization during synthesis or processing at a temperature above 260 °C for more than 1 h. As a result, the *cis*/*trans* ratio can reach values of 34–66%. To check if isomerization occurred during the polymerization process, we calculated the percentage of *cis* 1,4-cyclohexandicarboxylate subunits. We found comparable values for the two polymers: 4% for the homopolymer and 5% for the copolymer (Table 1). According to these data, isomerization from the *trans* conformation to the *cis* one occurred only to a small extent during the synthesis.

The molecular weights (M_n_) and dispersity indexes (Ð) of the two polyesters, obtained through GPC analysis, are listed in Table 1. In both cases, the molecular weights are high and similar, with narrow Ð values, indicating a proper control over polycondensation reaction, in line with previous studies [10,15].

**Figure 4:**
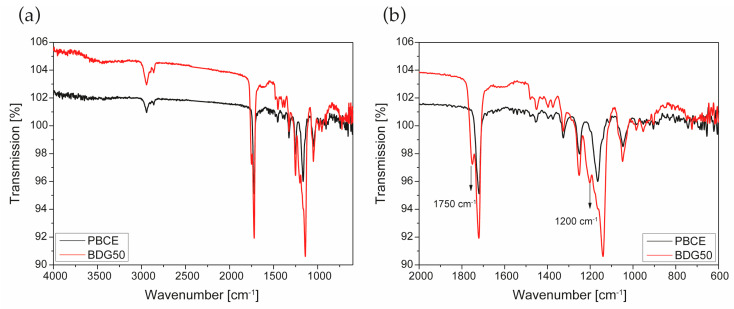
FT-IR spectra of PBCE and BDG50 scaffolds in the 4000–600 cm^−1^ (**a**) and 2000–600 cm^−1^ (**b**) frequency range.

Figure 4 shows the infrared spectra in ATR mode of the PBCE and BDG50 scaffolds in the 4000–600 cm^−1^ (a) and 2000–600 cm^−1^ (b) frequency ranges. The ATR-FTIR spectra of the polymer and copolymer show the C-H aliphatic stretching vibrations in the 2800–3000 cm^−1^ region and the OH stretching of inter- and intra-molecular bonding of hydroxyl groups in the 3100–3600 cm^−1^ range [33]. 

In addition, all samples display a strong absorption band in the 1700–1750 cm^−1^ region (Figure 4b) due to the C=O carbonyl stretching vibration of the PBCE at 1720 cm^−1^. A second band, less intense, is evident in the copolymer (BDG50) at 1750 cm^−1^, due to the copolymer chemical structure of the saturated aliphatic esters [34]. Lastly, the fingerprint region (1300–900 cm^−1^) of the copolymer is characterized by the following main modes with maximum absorbance at 1250, 1203, 1165, and 1140 cm^−1^, due to the C-C and C-O stretching region. The fingerprint area contains alternating spectral peaks that are very sensitive to the crystallinity changes and contains valuable information on the crystalline and amorphous region in the polymer and copolymer-based samples. In particular, the in-plane C–O–C ring stretching at 1165 cm^−1^ present in the PBCE scaffold reduces the intensity in the copolymer scaffold while increasing the corresponding peak at 1140 cm^−1^. Compression-molded films show similar FT-IR spectra, indicating that the electrospinning process does not affect the chemical composition of the polymer and copolymer used and does not degrade the chemical structure of the materials.

#### 3.1.2. Morphological Characterization

**Figure 5:**
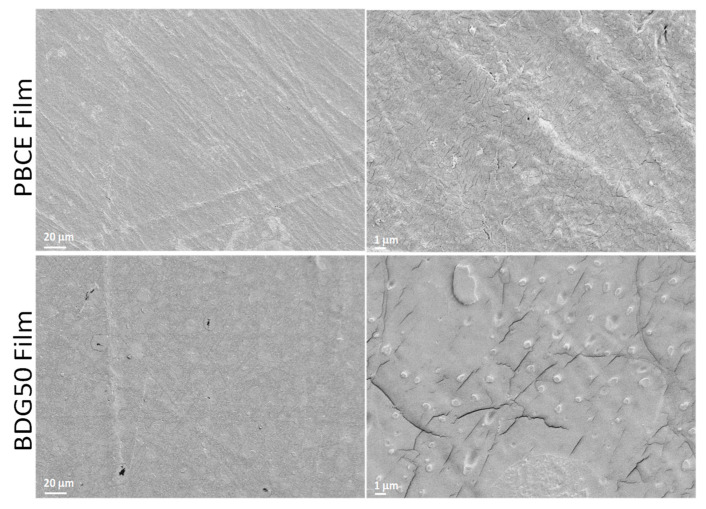
FESEM images of PBCE and BDG50 film surfaces at different resolutions.

**Figure 6:**
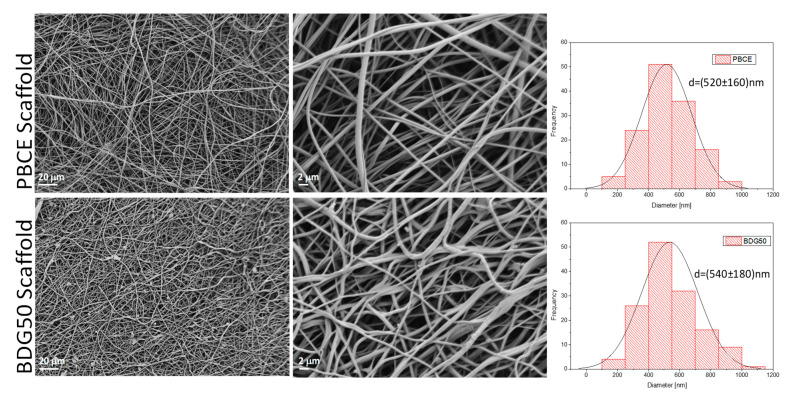
FESEM images of PBCE and BDG50 electrospun mat surfaces at different resolutions and the corresponding fiber diameter distribution.

Figure 5 and Figure 6 show the FESEM micrographs of the PBCE and BDG50 copolymer film surfaces and electrospun mats, respectively. The PBCE and BDG50 film surfaces appear smooth at different magnifications, underlining the efficiency of the copolymerization and melting process technique applied during the production phase. In previous research, it was observed that PBCE appeared with porous surface microstructure because of the solvent casting process applied [13]. This behavior was not observed because the sample was produced using a melt process instead of the solvent-casting technique. The FESEM images of the PBCE films have an even, compact, and smooth surface with evidence of parallel lines, while the copolymer films have rougher surface morphology (Figure 5). The FESEM images of the BDG50 copolymer film surface show the presence of a microphase separation; an array of circular microdomains of around 10 µm in diameter is visible. The resulting microdomains are spontaneously formed on the surface of copolymer films without templating, induced by the processing and by the copolymer structure (Figure 5).

The morphology of scaffold fibers, after the electrospinning procedures of the PBCE polymer and BDG50 copolymer, was investigated using FESEM, and related fiber diameter distribution is reported in Figure 6. The diameters distributions, as well as the mean diameters, were discussed to evaluate the dimension and homogeneity of the polymer and copolymer fibers. The FESEM images showed that all the randomly oriented fibers are cylindrical in shape and defect-free, since no beaded or ribbon-like fibers were observed. All these electrospun mats consisted of uniform, smooth fibers free of defects forming a porous, highly interconnected architecture. The BDG50 electrospun copolymers show comparable homogenous fibers with respect to the PBCE homopolymer. The reported measurements permit us to calculate an average fiber diameter of (520 ± 160) nm and (540 ± 180) nm for PBCE and BGD50, respectively. Collectively, these results demonstrated that the obtained electrospun fibers have a narrow size distribution and a comparable fiber size.

#### 3.1.3. Surface Hydrophobicity/Hydrophilicity Characterization

**Figure 7:**
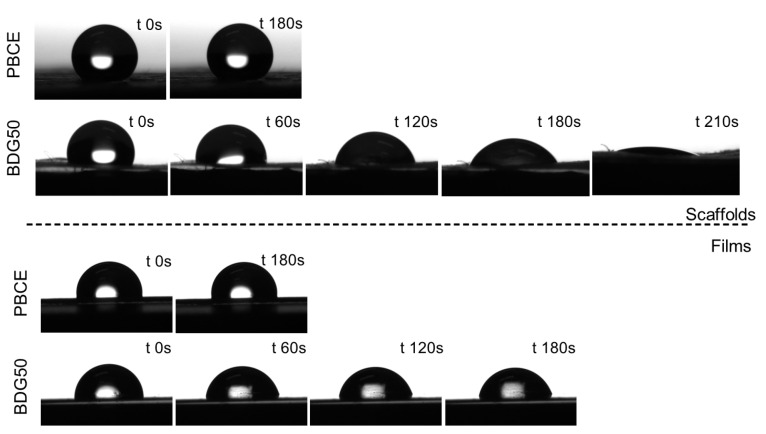
Pictures of water drops on PBCE and BDG50 scaffolds and films at different time points.

We performed the surface characterization using static water contact angle (WCA) measurements. The WCA values are reported in Table 1, while pictures of the drops poured on the scaffold and film surfaces at different time points (from 0 up to 210 s after deposition) are displayed in Figure 7. The PBCE homopolymer is hydrophobic, with WCA values of 100° for the film and 140° for the scaffold. In both cases, the drop shape did not change after deposition. Conversely, the BDG50 turned out to be less hydrophobic, with a WCA value of 91° for the film and 115° for the scaffold (Table 1, Figure 7), due to the presence of the ether oxygen atoms along the main chain. An evolution of drop shape was also recorded on the copolymer, with WCA values becoming progressively lower, reaching a value of 77° 3 min after deposition on the film surface. This measurement could not be performed on the scaffold due to the swelling of the surface in contact with water. Moreover, 210 s after deposition, an almost complete sorption of the water drop into the electrospun scaffold could be observed (Figure 7). This behavior is still caused by the number of BDG co-units, which improves material hydrophilicity. The highly porous surface morphology, compared to the continuous morphology of the film, helps improve the hydrophilicity.

#### 3.1.4. Thermal Characterization

**Figure 8:**
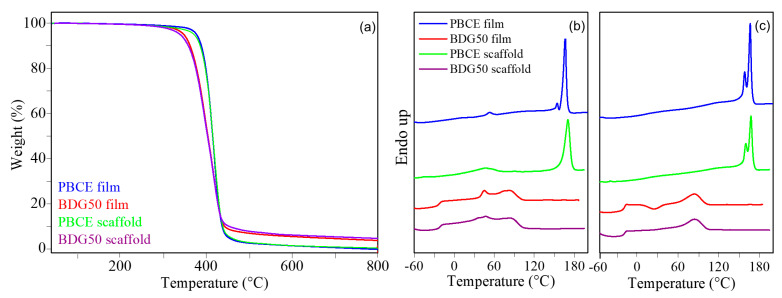
Calorimetric measurements were carried out on PBCE and BDG50 films and scaffolds (**a**) TGA, (**b**) I scan DSC curves, and (**c**) II scan DSC curves.

Thermal stability was evaluated using TGA measurements. The thermograms in Figure 8a show that both the films show high thermal stability, with a T_onset_ above 390 °C and T_max_ of 418 °C in both cases (Table 1). Weight loss occurred in one step and was complete for the PBCE. In the BDG50 film and scaffold, a small char residue of about 5% was measured. The copolymer started degrading at a slightly lower temperature with respect to the PBCE homopolymer due to the ether oxygen atoms, which favor thermos-oxidative processes [35,36]. If the scaffolds are compared to the relative films, no differences can be observed, indicating no residual solvents were trapped in the mats.

Differential scanning calorimetric analysis (DSC) revealed that in the first heating scan (Figure 8b), all films are semicrystalline, the DSC traces showing an endothermic baseline deviation associated with glass-to-rubber transition and a melting endotherm peak at higher temperature. If the BDG50 film is compared to the PBCE one, a decrease in both melting temperature, T_m_, and melting enthalpy, ∆H_m_, can be observed (Table 1) because of copolymerization. Indeed, the ether oxygen atoms in the BDG co-units are responsible for a decrease in the crystallization capability of BCE moieties (lower ∆H_m_) [10,12] together with the formation of less perfect crystals, which melt at lower temperatures. The higher crystallizing ability of the PBCE is also evidenced by the very low endothermal shift of baseline related to the glass transition phenomenon, which is not practically detectable (see Table 1 and Figure 8b). The PBCE and BDG50 scaffolds have DSC profiles similar to those of the films, although the PBCE scaffold showed a slight increase in ∆H_m_ due to the effect of solvent-induced crystallization, which occurred during electrospinning.

A further heating scan was performed after rapid cooling from the molten state (II scan). From the relative DSC traces (Figure 8c), the glass transition temperatures (T_g_) of films, measured after quenching, decrease from 15 °C for PBCE to −20 °C for BDG50. The measured ∆c_p_ value for the copolymer is higher than that of the homopolymer, proving that the BDG50 film contains a higher amount of amorphous phase. This is due to the enhanced chain flexibility conferred by the ether oxygen atom in the BDG co-unit with respect to the rigid aliphatic ring of BCE moieties [10,15]. For each polymeric material, the glass-to-rubber transition is quite similar for the films and the scaffold, although the difference between the ∆c_p_ of the two scaffolds is more modest. The rapid cooling from the melt was not effective in blocking macromolecular chains in a complete amorphous phase, as evidenced by the melting endothermic phenomenon present in all II scan traces. In detail, for the PBCE, the DSC profiles of the film and scaffold are quite similar, with multiple melting peaks indicating melting–crystallization–melting phenomena during the heating scan. Conversely, if the BDG50 is considered, a different crystallization capability can be observed by comparing the film and scaffold. Indeed, in the calorimetric curve of the scaffold, only the endothermic transition related to the melting of the crystalline phase is present. Conversely, in the DSC trace of the film, an exothermic phenomenon is present between T_g_ and T_m_, indicating that the quenching procedure was more effective and the macromolecular chains acquire enough energy and have enough mobility to rearrange in an ordered crystalline structure, which melts at higher temperatures. However, even the copolymeric film is semicrystalline, being ∆H_cc_ < ∆H_m_.

#### 3.1.5. Mechanical Characterization 

**Figure 9:**
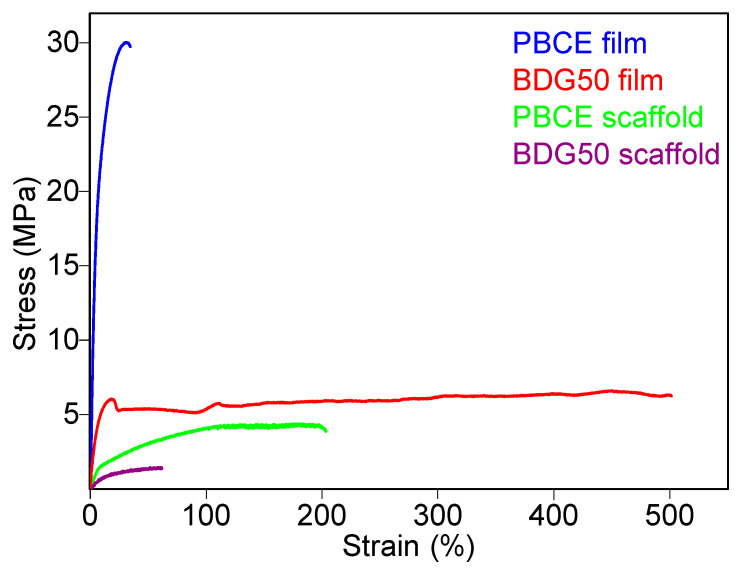
Stress–strain curves of PBCE and BDG50 films and scaffolds.

The mechanical properties of the PBCE and BDG50 scaffolds and films were investigated and compared. All samples were subjected to tensile measurements, knowing that cell viability and response are strictly related to the mechanical characteristics of the substrate onto which they are cultured [37]. The stress–strain curves are shown in Figure 9, while the corresponding mechanical data (elastic modulus, E, stress at break, σ_B_, and deformation at break, ε_B_) are listed in Table 1. From a comparison of the scaffolds and films, the PBCE is characterized by the typical behavior of brittle and rigid material, with a very low deformation at break together with a high elastic modulus. In contrast, the copolymer BDG50 films are much more flexible (reduced stiffness) because of the presence of ether oxygen atoms. Their elastic modulus and stress at break decrease about 5 times, from 560 to 94 MPa and from 27 to 6 MPa. A congruent trend is observed for elongation to break, which increases more than 10 times, from 40% for the PBCE to over 490% for the BDG50. Since both the investigated polymers display a soft amorphous phase (T_g_ values are in all cases below room temperature), the different mechanical response can be ascribed to the lower crystallinity degree of the copolymer (Table 1), due to its lower symmetry, because of the presence along the macromolecular chain of ether oxygen atoms. Last, for the BDG50, the yielding phenomenon can be observed after an elongation of about 20%.

With regard to the scaffolds obtained from the polyesters under investigation, very different behaviors can be observed compared to those of the relative films. In detail, for the PBCE homopolymer, a decrement in E (which becomes about 30 times lower) and σ_B_ (which decreases from 27 to 4 MPa) together with an increase in ε_B_ of about 5 times is observed when the compression molded film is compared with the electrospun mat. 

A decrease in both E and σ_B_ was also observed for the copolymer, with values reducing from 94 to 8 MPa and from 6 to 1.5 MPa, respectively. As expected, the BDG50 scaffold has an elastic modulus lower than the PBCE mat, with a value very close to those of some soft tissues, such as cartilage [38,39,40]. The elongation at break decreased with respect to the film’s value. We speculated that the porosity created by the interconnected fibers behaves as microscopic defects, which negatively affect the maximum elongation reached under tensile testing. 

Lastly, electrospinning allowed us to obtain materials that do not undergo yielding, unlike to what was observed for both films.

Collectively, the chemical–physical characterization results indicated that (i) the PBCE scaffold was more hydrophobic than the BDG50 copolymer, and both are more hydrophobic than their film counterparts; (ii) the PBCE and BDG50 scaffolds have T_g_ values similar to those of their relative films; and (iii) the PBCE scaffold was stiffer than the BDG50 copolymer, but both were significantly less stiff than their film counterparts (E: PBCE film >> BDG50 film >>> PBCE scaffold > BDG50 scaffold). Thus, the 3D structure has a critical impact on the characteristics of the homopolymer and copolymer. 

### 3.2. Biological Evaluation

Next, we investigated whether the PBCE and BDG50 scaffolds were suitable for biological applications.

#### 3.2.1. Protein Adsorption 

First, we measured protein adsorption to assess the protein-binding capabilities of the PBCE and BDG50 scaffolds compared to films. Protein adsorption is the first indicator revealing biological systems’ responses to materials [40]. The experiments were carried out according to our protocol [16,20], with 2 mg/mL of purified BSA, 2% and 10% of FBS, and 5 mg/mL of plasma at two different time points of incubation, 30 min and 24 h.

**Figure 10:**
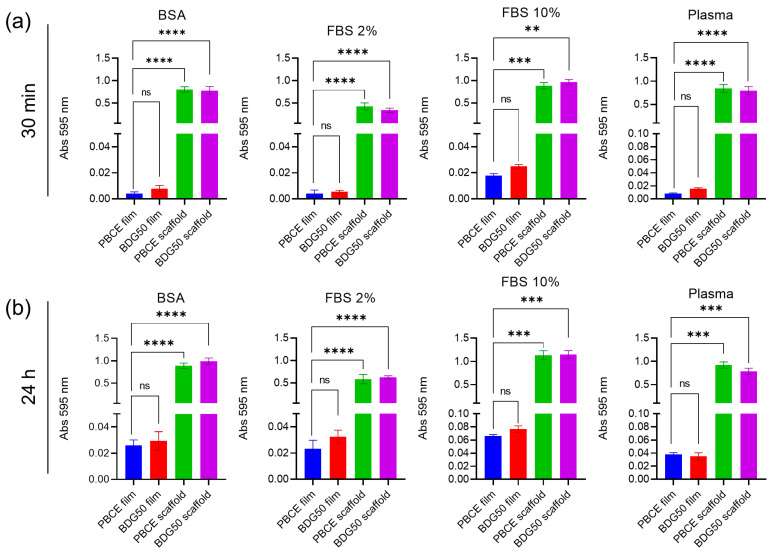
Adsorbed protein on PBCE and BDG50 films and scaffolds. (**a**) Total adsorbed proteins (absorbance 595 nm) from BSA, FBS, and plasma after 30 min at 37 °C and (**b**) after 24 h at 37 °C incubation. Protein values adsorbed in PBCE film are shown in blue, in BDG50 film in red, in PBCE scaffold in green, and in BDG50 scaffold in purple. The data are expressed as percentages of PBCE films and are the mean + SD of three independent experiments. The significance of the differences is indicated as follows: ** *p* < 0.01, *** *p* < 0.001, **** *p* < 0.0001; ns: not significant.

No significant changes were observed in the adsorption of BSA, FBS, and plasma in the PBCE and BDG50 scaffolds after 30 min and 24 h of incubation, indicating that the homopolymer and copolymer scaffolds had the same surface capability toward different proteins and that 30 min incubations were sufficient to reach the saturation of the adsorption potentials of electrospun mats (Figure 10). Significant increases in the adsorption capability of the above proteins were observed in the PBCE and BDG50 films after 24 h incubation compared to the corresponding films at 30 min (Figure 10). Of note, the PBCE and BDG50 scaffolds have a higher protein adsorption capability than the PBCE and BDG50 films. This effect is due to the higher roughness, porosity, different topography, nanostructured fibers, and the higher surface area of the scaffolds that is accessible for protein interaction in the electrospun structure. These findings agree with other studies that demonstrated how surface roughness and porosity influence the adsorption capability of proteins (e.g., BSA) in artificial polymer films [41,42,43]. These results are indicators of the potential cell protein–surface interactions on scaffolds and films of PBCE and BDG50.

#### 3.2.2. PBCE and BDG50 Scaffolds Are Suitable for adult human Multipotent Mesenchymal/Stromal Cell Culture 

Next, we evaluated the biocompatibility of the PBCE and BDG50 scaffolds toward hBM-MSCs. In comparison, experiments were conducted by seeding hBM-MSCs on PBCE and BDG50 films [14].

**Figure 11:**
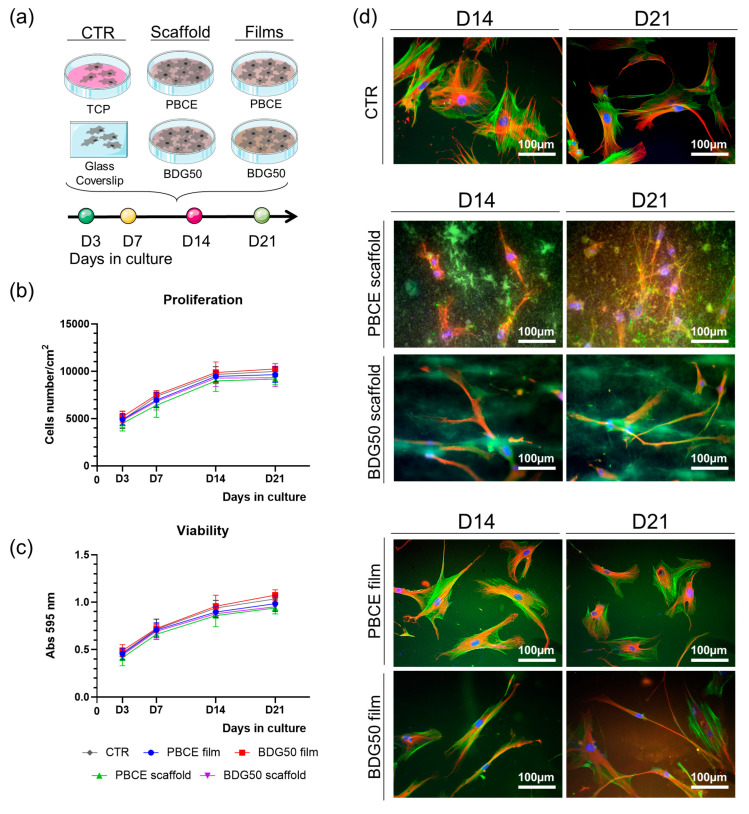
Culture of hBM-MSCs on PBCE and BDG50 film, PBCE scaffold, and BDG50 scaffold. (**a**) Schematic representation of the experimental plan. (**b**) hBM-MSC proliferation on PBCE and BDG50 films and scaffolds and in canonical culture conditions. Data are reported as the mean ± SD of three independent experiments. (**c**) MTT assay of cells on PBCE and BDG50 films and scaffolds and in canonical culture conditions (TCP). Data are reported as mean ± SD of three independent experiments. (**d**) Representative fluorescence images of nuclei (blue), F-Actin (green), and β-Tubulin (red) of cells on PBCE and BDG50 films, PBCE and BDG50 scaffold, and CTR. Scale bar = 100 μm.

hBM-MSCs were cultured on PBCE and BDG50 on scaffolds and films (Figure 2 and Figure 11a), in the growth medium, for a period of 21 days (D21). TCP/GC was used as the experimental control (CTR).

Cell proliferation and viability were assessed at D3, D7, D14, and D21 (Figure 11b,c). Comparable rates of cell proliferation were observed in hBM-MSCs on the PBCE and BDG50 scaffolds and films with regard to CTR cultures (Figure 11b). No cytotoxicity was observed in cells on the PBCE and BDG50 scaffolds, as shown by the similar mitochondrial dehydrogenase activity curves to those of the control systems (Figure 11c). Confirming previous work [14], no cytotoxicity was observed in cells on the PBCE and BDG50 films (Figure 11c). These findings indicated the biocompatibility of the PBCE and BDG50 scaffolds for hBM-MSCs long-term cultures and further validate our previous results on the biocompatibility of PBCE and BDG50 polymers for hBM-MSCs [14]. 

**Figure 12:**
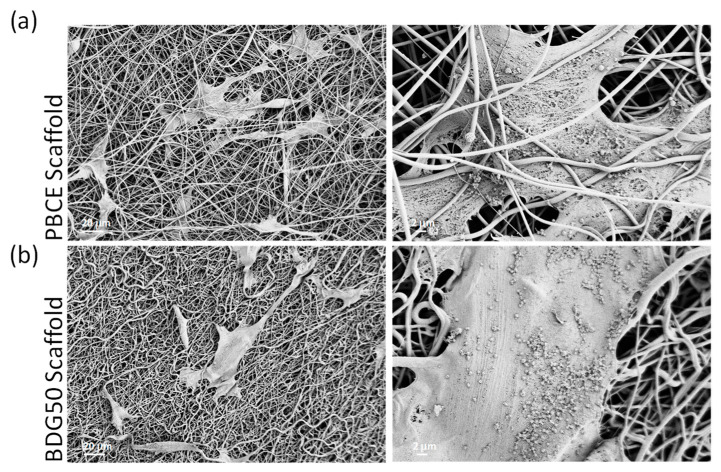
FESEM images of stem cells adhered on PBCE (**a**) and BDG50 (**b**) electrospun mat surfaces at different resolutions.

We also evaluated the effect of the scaffold on the cell cytoskeleton architecture (Figure 11d). The immunostaining of F-Actin and βTubulin revealed the significant elongation of hBM-MSCs on the PBCE and BDG50 scaffolds (Figure 11d). Although the 3D structure makes the microcopy visualization difficult, these cells’ morphology is different from the fibroblast-like morphology of BM-MSCs in canonical culture conditions (Figure 11d, CTR, representative images) [16,20]. Both F-Actin microfilaments (green) and microtubules (red) revealed the long extension of the cell protrusions and mainly the cell distribution into the PBCE and BDG50 scaffolds (Figure 11d, scaffolds, representative images). Interestingly, the cell elongation and positioning were more emphasized on the BDG50 scaffold compared to the PBCE one, suggesting the influence of the properties acquired with the copolymerizing on the cell response to the scaffold fibers (Figure 11d, scaffolds, representative images). The lengthening and remodeling of the cytoskeleton structure were observed in the long-term culture, indicating the new stable cell morphology (Figure 11d, scaffolds, representative images). The interaction of hBM-MSCs on the PBCE and BDG50 scaffolds was also evident in a short-term culture (Figure 12, representative images). 

FESEM images revealed cells with an elongated stretched morphology in all directions following the fiber configurations and confirming the above results (Figure 10d). Interestingly, the electrospun porous and interconnected microstructure obtained with the PBCE homopolymer permitted the multipotent cells to penetrate the polymer mats. The images also indicated a higher amount of cell infiltration in mats fabricated with the PBCE compared with those produced with the copolymer BDG50, suggesting a better capacity of hBM-MSCs to interact with the homopolymer compared to the copolymer (Figure 12, representative images).

The effects of the copolymerization were also emphasized in hBM-MSCs on the PBCE and BDG50 films (Figure 11d films, representative images). According to our previous results, hBM-MSCs on PBCE homopolymer film have a comparable morphology to those on CTR (Figure 11d films, representative images; [12,14]), whereas the inclusion of DG co-units in PBCE films caused a change in the architecture of the cytoskeleton of the hBM-MSCs. However, the cell elongation was the new cell morphology (Figure 11d films, representative images), suggesting that the change was not dependent on the 2D or 3D structure of the support but was related to the change in mechanical properties (homopolymer and copolymer films are stiffer than the relative scaffolds).

The overall findings demonstrated that the PBCE and BDG50 scaffolds are as suitable for long-term stem cell culture as PBCE and BDG50 films. 

## 4. Conclusions

In the present paper, we have designed and produced a new 3D scaffold with targeted properties and microstructure suitable for long-term multipotent/progenitor cell culture. We have exploited different tools, used synergistically—on the one hand, an appropriate chemical modification of a previously investigated and biocompatible synthetic cycloaliphatic polyester (PBCE), which represents our reference polymer, and on the other, the processing of the material object of this study—for the realization of a structure with high and controlled roughness/porosity (scaffolds) obtained through the well-known electrospinning technique. The chemical modification of PBCE, consisting of the introduction into its chain of a consistent number of ether linkages, made it possible to significantly reduce the stiffness of the material, simultaneously increasing its hydrophilicity due to the intrinsic chemical characteristics of this type of bond, conferred by the presence of the small and highly electronegative oxygen atoms. The realization of the highly porous structure with an interconnected pore network permitted us to further decrease the polymer elastic modulus and thus obtain a softer material and contributed to offsetting the increment in the material hydrophilicity of the BDG50 scaffold. The PBCE and BDG50 scaffolds have a higher protein adsorption capability compared to their film counterparts. The PBCE and BDG50 scaffolds were biocompatible and suitable for hBM-MSC long-term cultures. However, likely due to the 3D nanostructures of the PBCE and BDG50 electrospun scaffolds, the FESEM micrographs displayed that cells colonized the structure of the PBCE better than BDG50. It is noteworthy that hBM-MSCs responded to the PBCE and BDG50 scaffolds differently to the PBCE and BDG50 films. On the PBCE scaffolds, hBM-MSCs remodeled the cytoskeleton architecture and acquired a new elongated morphology that was confirmed and enhanced on the BDG50 scaffolds. The new cell morphology recapitulated those of hBM-MSCs on the BDG50 films. We suggest that the significantly reduced stiffness in the PBCE and BDG50 scaffolds might influence the remodeling of the cell shape, as on the BDG50 films. These results agree with our recent findings showing the role of stiffness in the shape of human multipotent mesenchymal/stromal cells [14] and are correlated with other findings about the impact of stiffness on cell morphology [44,45].

In conclusion, we have demonstrated the highly-tuned potential of the PBCE polymer to be manipulated for generating films and, foremost, 3D scaffolds. Furthermore, we have validated how the same polymer (PBCE) acquired new chemical–physical properties depending on the processing technique (e.g., solvent casting vs. compression molding [12]; films vs. scaffolds). Finally, we have highlighted the role of the copolymerization of BDG units in emphasizing the homopolymer PBCE, even for biological applications. 

## Figures and Tables

**Table 1:** Molecular (^1^H-NMR and GPC), surface (WCA immediately after deposition (WCA T0) and 180 s after deposition, WCA 180 s), thermal (DSC and TGA), and mechanical (stress–strain measurements) characterization data of PBCE homopolymer and BDG50 copolymer.

	PBCE		BDG50	
Molecular Characterization				
**M_n_ (g/mol)**	50,300		47,500	
Ð	1.7		1.5	
BCE feed (%)	100		50	
BCE actual (%)	100		52	
*Cis* (%)	4		5	
	***scaffold***	***film***	***scaffold***	***film***
WCA (°) T0	140 ± 2	100 ± 3	115 ± 1	91 ± 2
WCA (°) 180 s	140 ± 2	100 ± 3	-	77 ± 2
**Thermal Characterization**				
**Thermogravimetric Analysis**				
	***scaffold***	***film***	***scaffold***	***film***
T_onset_ (°C)	392	395	364	366
T_max_ (°C)	418	418	417	417
**Differential Scanning Calorimetry**				
***First scan***				
	***scaffold***	***film***	***scaffold***	***film***
T_g_ (°C)	-	-	−21	−22
ΔC_p_ (J/g°C)	-	-	0.270	0.299
T_m_ (°C)	170	166	47 86	45 84
ΔH_m_ (J/g)	36	29	3 18	4 16
***Second scan***				
	***scaffold***	***film***	***scaffold***	***film***
T_g_ (°C)	13	15	−19	−20
ΔC_p_ (J/g°C)	0.221	0.322	0.277	0.426
T_cc_ (°C)	-	-	25	-
ΔH_cc_ (J/g)	-	-	5	-
T_m_ (°C)	160 167	158 166	84	84
ΔH_m_ (J/g)	8 16	8 21	15	15
**Mechanical Characterization**				
	***scaffold***	***film***	***scaffold***	***film***
E (MPa)	17 ± 3	560 ± 19	8.0 ± 1.4	94 ± 6
σ_B_ (Mpa)	4.0 ± 0.7	27 ± 2	1.5 ± 0.4	6.0 ± 0.5
ε_B (%)_	190 ± 27	40 ± 5	61 ± 4	494 ± 10

-, not detectable.

## Data Availability

This study was conducted in accordance with the Declaration of Helsinki. Mesenchymal cells were isolated from waste samples from the surgery of an adult donor, who gave written consent. Ethical review and approval were waived for this study because procedures were sporadic and not associated with a specific project.
